# Radiation Induces Apoptosis and Osteogenic Impairment through miR-22-Mediated Intracellular Oxidative Stress in Bone Marrow Mesenchymal Stem Cells

**DOI:** 10.1155/2018/5845402

**Published:** 2018-08-12

**Authors:** Zhonglong Liu, Tao Li, Si'nan Deng, Shuiting Fu, Xiaojun Zhou, Yue He

**Affiliations:** ^1^Department of Oral Maxillofacial & Head and Neck Oncology, Shanghai Ninth People's Hospital Affiliated to Shanghai Jiao Tong University School of Medicine, Shanghai 200011, China; ^2^Department of Orthopedics, Shanghai Ninth People's Hospital Affiliated to Shanghai Jiao Tong University School of Medicine, Shanghai 200011, China; ^3^Department of Stomatology, Central Hospital of Min-Hang District, Shanghai 201109, China

## Abstract

Bone marrow mesenchymal stem cells (BMSCs) were characterized by their multilineage potential and were involved in both bony and soft tissue repair. Exposure of cells to ionizing radiation (IR) triggers numerous biological reactions, including reactive oxygen species (ROS), cellular apoptosis, and impaired differentiation capacity, while the mechanisms of IR-induced BMSC apoptosis and osteogenic impairment are still unclear. Through a recent study, we found that 6 Gy IR significantly increased the apoptotic ratio and ROS generation, characterized by ROS staining and mean fluorescent intensity. Intervention with antioxidant (NAC) indicated that IR-induced cellular apoptosis was partly due to the accumulation of intracellular ROS. Furthermore, we found that the upregulation of miR-22 in rBMSCs following 6 Gy IR played an important role on the ROS generation and subsequent apoptosis. In addition, we firstly demonstrated that miR-22-mediated ROS accumulation and cell injury had an important regulated role on the osteogenic capacity of BMSCs both in vitro and in vivo. In conclusion, IR-induced overexpression of miR-22 regulated the cell viability and differentiation potential through targeting the intracellular ROS.

## 1. Introduction

The delivery of radiotherapy is often required in oral and maxillofacial regions to serve as a major or an adjuvant therapy for malignancies. In addition to the effective control of local disease, damaging normal bone and soft tissues within the radiation field is inevitable. Radiation-induced skeletal system injury is characterized by the destruction of osteocytes, a deficiency of osteoblasts and osteoid, bone marrow fibrosis, a lack of bone marrow mesenchymal stem cells (BMSCs), and even osteoradionecrosis [1, 2]. This complication may contribute to the loss of metabolic equilibrium in bone formation.

Ionizing radiation (IR) may sensitize the bone marrow cells and osteoblasts to apoptogens and induce the apoptotic process, thus causing profound ramifications for osteogenic function and further bone formation [3]. BMSCs are one of the major types of progenitor cell, which hold the capability to differentiate into multilineage cells, including osteoblasts, and maintain the homeostasis with osteogenesis. The topic of whether mesenchymal stem cells (MSCs) are radiosensitive or radioresistant is still controversial. Some scholars supported that MSCs show considerably high radioresistance both in vitro and in vivo [4–7], while these MSCs may be different from those derived from bone. Others verified that BMSCs were sensitive to X-ray or *γ-*radiation, and a small portion of these cells developed apoptosis following exposure to different dosages [8–10]. Accordingly, radiation response of MSCs is a complicated biological process, and it may depend on cell-to-cell variations and resource of radiation, thus triggering different signals or mechanisms to determine the cell fate.

IR leads to the production of oxygen-derived free radicals and reactive nonradical molecules, so-called reactive oxidative species (ROS), which may impose indirect damage onto cells when this excessive oxidative stress is beyond the scavenge ability of antioxidant detoxification systems [11, 12]. Radiation-induced ROS generation has also been proven both in vitro and in vivo studies [13, 14]. IR and UV are the most important physical factors that trigger the generation of prooxidant compounds and the production of oxidative stress [15]. Moderate ROS is deemed as an indispensable stress or molecules involved in the normal physiological reaction, whereas enhanced or excessive ROS may influence the cell survival or death fate, including proliferation and apoptosis [16]. ROS participates in cellular signal transduction and acts as the main regulator in the pathways mediating apoptosis, such as mitochondrial pathway, death receptor pathway, and endoplasmic reticulum pathway [17].

MicroRNAs (miRNAs) belong to noncoding RNAs, which are initially transcribed in the nucleus by RNA polymerase II, and have negative regulation of mRNA through degradation or posttranscriptional inhibition via binding to the 3′-untranslated region (3′-UTR) of target mRNAs [18]. In recent years, miRNAs have been verified as multifunctional genes involved in the cell cycle, survival and death, proliferation, differentiation, and so on [19]. By using miRNA microarrays, several publications have validated the upregulation of miR-22 after UV or IR within hours, suggesting that gene regulation of miRNAs occurred before the transcriptional responses of mRNA [20–23]. Other scholars found that the expression of miR-22 in the human lymphoblast cell line TK6 following radiation (2 Gy) exhibited two peaks of induction (8 h and 24 h post-IR) and was fluctuant with crests and troughs [24]. However, the biological function of this expression change has not been elucidated.

Through a review of the literature, miR-22 is defined as a multifunctional biomolecule involved in proliferation, cell survival, cell cycle, tumor invasiveness, and cardioprotection [22, 25–27]. Recent studies also demonstrated that miR-22 participated in the regulation of total intracellular ROS or mitochondrial ROS regeneration [28–30]. However, it is not clear whether radiation-induced miR-22 expression has a role on the regulation of IR-induced production of ROS and cellular apoptosis and subsequently osteogenic impairment. The current study was designed to elucidate the relationship of miR-22 with ROS and apoptosis, as well as the osteogenesis of BMSCs following radiation.

## 2. Material and Methods

### 2.1. Reagents and Chemicals

Annexin V-FITC/PI detection kit was purchased from BD Biosciences (San Jose, CA, USA). Fluorometric Intracellular ROS Kit and N-acetyl-L-cysteine (NAC) were from Sigma-Aldrich (St. Louis, USA). Information of primary antibodies is listed as follows: anti-Runx2 (1 : 500, Abcam), anti-Osterix (1 : 500, Abcam), anti-OPN (1 : 1000, Abcam), anti-BSP (1 : 1000, CST), anti-OCN (1 : 500, Abcam), anti-NADPH oxidase 4 (NOX4) (1 : 500, Abcam), anti-SOD2 (1 : 500, Abcam), anti-Caspase-3 (1 : 1000, CST), and anti-GAPDH (1 : 5000, Bioworld Technology Inc., USA). ALP staining and alizarin red were both from Cyagen (Guangzhou, China). For semiquantitative analysis, p-nitrophenyl phosphate (p-NPP) and 10% cetylpyridinium chloride were from Sigma-Aldrich (St. Louis, USA). Alexa Fluor 488 conjugated was from Jackson ImmunoResearch (USA). DAPI was from Sigma-Aldrich (St. Louis, USA). For scaffold fabrication, gelatin, carboxymethyl chitosan, 1-ethyl-3-(3-dimethylaminopropyl)carbodiimide hydrochloride (EDC), and N-hydroxysuccinimide (NHS) were all purchased from Aladdin (Shanghai, China). Calcein AM (4 mM) and Lipofectamine 3000 were from Thermo Fisher Scientific (USA).

### 2.2. Rat BMSC (rBMSC) Isolation and In Vitro Culture

The current study was approved by the Ethics Committee of Shanghai Ninth People's Hospital. Male 4-week-old Sprague-Dawley rats were obtained from the Department of Experimental Animals in our institution. The SD rats were sacrificed through cervical dislocation and then were sterilized in 75% ethanol for approximately 10 min. The bilateral tibias were dissected free of muscle and connective tissue and were immersed into sterile PBS immediately. Both ends of the tibia were cut to expose the marrow cavity. A 1 ml syringe (BD Biosciences, San Jose, CA, USA) was used to repeatedly flush the bone marrow into a 10 cm dish with complete media. The cell suspension was then centrifuged at 1000 rpm for 5 min. We resuspended the cell sedimentation with complete medium containing 10% fetal bovine serum (Gibco, Thermo Fisher Scientific, USA), *α*-modified Eagle's medium (HyClone, USA), and 1% penicillin-streptomycin (HyClone, USA). This suspension was then filtered with a 70 *μ*m cell strainer (BD Biosciences, San Jose, CA, USA) and was seeded into a 25 cm^2^ flask for incubation at the condition of 37°C and 5% CO_2_. After 48 h incubation, floating cells were removed, and fresh complete medium was added. Medium change was performed every 3 days. Once the cell confluence reached 80%–90%, cell expansion (1 : 3) was taken into consideration. Briefly, cells were treated with 1 ml of 0.25% EDTA-trypsin (Gibco, Thermo Fisher Scientific, USA) for 1 min. Then, complete medium was added to neutralize the trypsin. The novel cell suspension was cultured in three flasks of 25 cm^2^. For osteogenic differentiation, cells at passage 3 with 80% confluence were induced under *α*-MEM supplemented with 10% FBS, 10 nM dexamethasone, 10 mM *β*-glycerol phosphate, and 50 *μ*g/ml ascorbic acid. Osteogenic induction medium was changed every 3 days.

### 2.3. Cellular Exposure to Ionizing Radiation (IR)

rBMSCs of the third passage were cultured in 6 cm *φ* dishes with complete medium and then were moved to a radiotherapy room when cells reached confluence at 80%. IR was performed in cells using 6 MeV (Precise Treatment System, Elekta, Swedish) with a dosage of 6 Gy and a dose rate of 600 Mu. Cells were then moved back to the incubator for continuous culture before collecting samples.

### 2.4. miRNA Isolation and Real-Time PCR Analysis

Total miRNA was extracted using the miRcute miRNA Isolation Kit (Tiangen Biotech, Beijing, China), and total miRNA was reverse-transcribed using miRcute miRNA First-Strand cDNA Synthesis Kit (Tiangen Biotech, Beijing, China). Briefly, Poly(A) was added to the 3′ end of miRNA, and then this production was reverse-transcribed using the oligo(dT)-universal tag to produce the first-strand cDNA. The relative miR-22 gene expression level was analyzed using miRcute miRNA qPCR Detection Kit (SYBR Green) (Tiangen Biotech, Beijing, China) in a 7300 Real-Time PCR system. U6 served as the endogenous normalization control. The fold change in miR-22 expression was determined by the comparative CT method 2^−∆∆CT^.

### 2.5. Lentiviral Vector Construction and Transduction

Plasmid vectors (pLenti-hU6-MSC-ubiquitin-EGFP-IRES-puromycin) were composed of rno-miR-22-NC, rno-miR-22, rno-miR-22-inhibitor-NC, and rno-miR-22-inhibitor and were obtained from GeneChem Technology Co., Ltd., China. Then, we transfected the 293T cells with plasmids shown above and Lipofectamine 3000 to produce the lentiviruses and collected the supernatant at 48 h after transfection. This supernatant with lentiviruses was then filtered and concentrated by using ultrafiltration. For the transfection procedure, rBMSCs were immersed in medium containing lentiviruses with 50 MOI, Opti-MEM, and 5 *μ*g/ml polybrene for 24 h. The transfected efficiency was evaluated through RT-PCR and fluorescence microscopy.

### 2.6. Cellular Apoptosis Assay

An Annexin V-FITC/PI detection kit was used to measure the apoptotic ratio of cells by flow cytometry according to the manufacturer's instruction. Briefly, 5 × 10^5^ cells (24 h after radiation, 0 or 6 Gy) were collected by trypsinization, washed with ice-cold PBS, and then resuspended in 300 *μ*l of 1x binding buffer containing 5 *μ*l Annexin V FITC, followed by dark incubation for 10 min at room temperature. PI (5 *μ*l) was added to each sample for coincubation for another 5 min. After incubation, 200 *μ*l of 1x binding buffer was added to further resuspend the cells, and at least 10,000 cells were measured on a BD FACS flow cytometer (FL1 and FL2 emission filter).

### 2.7. Measurement of Intracellular ROS Levels

rBMSCs were seeded on a 12-well plate in triplicates with a density of 2 × 10^4^ cells. After routine culture (with or without NAC) or lentiviral transfection, cells were exposed to 0 or 6 Gy IR, and analysis was performed at 24 h after X-ray treatment. For ROS staining, cells were incubated with a Fluorometric Intracellular ROS Kit for 45 min (5% CO2, 37°C), gently washed with PBS 3 times, and then imaged with the fluorescence microscope. For ROS level detection, the fluorescence intensity (*λ*ex = 640/*λ*em = 675 nm) of incubated cells was measured with a microscope. The results are shown as the mean fluorescence intensity of 2 × 10^4^cells ± SEM.

### 2.8. Western Blot Analysis

Whole cell lysates were acquired using RIPA lysis buffer and PMSF (1 mM) (Beyotime, China) by incubation on ice for 30 min. The protein concentration was detected using a BCA Protein Assay Kit (Pierce™, Thermo Fisher Scientific, MA, USA). Equal amounts (20 *μ*g/well) of protein samples were separated by SDS-PAGE (10%, 15%) and then transferred to PVDF (0.45 or 0.22 *μ*m) membranes (Millipore Corporation, MA, USA). The membranes were blocked in 5% BSA containing TBS for 1 h and then incubated with primary antibodies. After washing three times with TBST, the membranes were further incubated with HRP-tagged secondary antibodies for 1 h at room temperature. Finally, the protein bands were visualized by Odyssey V3.0 image scanning (LI-COR, Lincoln, NE, USA). The densitometric intensities of the individual protein bands were quantified using ImageJ software (Version 1.8.0), and the values were normalized to the GAPDH values for each sample.

### 2.9. Alkaline Phosphatase (ALP), Alizarin Red S (ARS) Staining, and Semiquantitative Analyses

rBMSCs were seeded onto 12-well plates at a density of 5.0 × 10^5^ cells. After routine culture or lentivirus transfection, cells were exposed to 0 or 6 Gy. Six hours after radiation, culture medium was replaced by osteogenic medium. On day 14, the cells were fixed in 4% paraformaldehyde, and ALP staining was performed according to the manufacturer's instructions. On day 21, after fixation in 90% ethanol for 20 min, cells were stained with alizarin red for 30 min at room temperature to detect matrix mineralization. Each experiment was repeated in triplicate. For ALP semiquantitative analyses, p-NPP was used as substrate and absorbance was measured at 405 nm in a microplate reader (Tecan Infinite M200, Switzerland). ARS staining was dissolved using 10% cetylpyridinium chloride, and the absorbance was read at 590 nm in Tecan M200. Finally, these data were normalized to the protein concentration of each sample.

### 2.10. Cellular Immunofluorescence

Cells were seeded onto confocal dishes (20 mm in diameter) at a density of 1 × 10^4^ cells/dish. Lentivirus transfections (miR-22-NC, miR-22, miR-22-inhibitor-NC, or miR-22-inhibitor) were performed when cells reached confluence at 30–50%. Twenty-four hours after transfection, cells were exposed to 6 Gy radiation. Osteogenic induction began at 6 h after X-ray treatment and lasted for 14 days. The samples were fixed in 4% paraformaldehyde for 30 min, permeabilized with 0.5% Triton X-100 for 20 min, and blocked with 2% BSA for 30 min, respectively. Next, primary antibodies of anti-Runx2 (10 *μ*g/ml, Abcam) and anti-Osterix (1 : 200, Abcam) were added to samples for incubation at 4°C overnight. The cells were then immersed in Alexa Fluor 488-conjugated secondary antibody for 1 h at RT. DAPI (Sigma-Aldrich, St. Louis, USA) was used to label cell nuclei for 10 min. The immunoreactive cells were visualized and captured using confocal microscopy (Leica TCS SP8, Germany). The ratio of positive cells in each sample was determined by dividing the number of immune-positive cells by the number of nuclei stained with DAPI in three random fields for each group.

### 2.11. Fabrication of Gelatin (G) and Carboxymethyl Chitosan (CMC) Scaffold

We obtained the CMC solution by adding 100 mg of powder to ultrapure water, which was then dissolved at 40°C. Subsequently, gelatin powder (1000 mg) was added to CMC solution through stirring for at least 1 h to obtain the G-CMC solution. The compound was then poured into the mold with different sizes, followed by frozen overnight (−20°C) and lyophilized for 48 h. Next, dried scaffolds were cross-linked by using EDC and NHS in a mixed solvent of acetone and water (volume ratio = 4 : 1) for 24 h at 4°C. The scaffolds were lyophilized and stored at −20°C. The surface morphology of the 3D scaffold was visualized and captured by SEM (TM-1000; Hitachi, Tokyo, Japan). The verification of scaffold constitution was applied via Fourier transform infrared spectroscopy (FITR).

### 2.12. Cell Adhesion in CLSM and SEM Imaging

Gelatin-chitosan scaffolds were immersed into 75% ethanol for 6 h and transferred to a 48-well plate with additional ethanol disinfection for 3 h. Sterile PBS with 1% penicillin-streptomycin was used to wash the residual ethanol for another 6 h. Finally, scaffolds were incubated with basal culture medium overnight at 37°C and 5% CO_2_. Cells with different treatments were seeded onto scaffolds at a density of 5 × 10^4^/well and were cultured for 5 days. Samples were then labeled with 4 mM calcein AM for 20 min and were visualized by confocal microscopy (FITC channel) after rinsing with PBS. Scanning electron microscopy (SEM) imaging was performed as previously described. Briefly, cell-seeded scaffolds were fixed in 2.5% glutaraldehyde for 2 h, then dehydrated in a graded ethanol series (100%, 90%, 80%, 70%, and 50%), and finally air-dried for 1 h. Gold-sputtered specimens were prepared using a JFC-1200 fine coater (JEOL, Tokyo, Japan) at 30 mA under high vacuum for 70 sec. The morphology and adhesion of the cells were visualized through SEM (TM-1000; Hitachi, Tokyo, Japan), which was manipulated at 15 kV under high vacuum mode.

### 2.13. Animal Surgical Procedure

Sprague-Dawley (SD) rats (8-week-old, female, weight: 160–200 g) were anesthetized and were cut on the scalp to make a 2 cm sagittal incision. Two critical-sized calvarial defects (CSDs) were created bilaterally on the scalps using a 5 mm-diameter trephine (Nouvag AG, Goldach, Switzerland). G-CMC (thickness: 1 mm, diameter: 5 mm) with or without cell adhesion was implanted to repair defects. Twenty-four rats were randomly allocated into the following groups: (1) G-CMC/BMSCs/Lenti-miR-22-NC (*n* = 6), (2) G-CMC/BMSCs/Lenti-miR-22 (*n* = 6), (3) G-CMC/BMSCs/Lenti-miR-22-inhibitor-NC (*n* = 6), (4) G-CMC/BMSCs/Lenti-miR-22-inhibitor (*n* = 6). Additionally, experimental groups were implanted on the right side and the control group was placed at the left side.

### 2.14. Microcomputed Tomography (Micro-CT) Analysis

The SD rats were sacrificed at 8 weeks after surgical procedure. The skull samples pretreated with 4% paraformaldehyde were then scanned using micro-CT (*μ*CT 80, Scanco Medical, Switzerland) with the slice thickness of 20 *μ*m and pixel matrix of 1024 × 1024. Subsequently, auxiliary histomorphometric software (Scanco Medical AG, Switzerland) was used to assess the three-dimensional structure of bone tissue at surgical fields. Other assessments, such as new bone volume relative to tissue volume (BV/TV) and the bone mineral density (BMD), trabecular number (Tb.N) were also concluded in the current study.

### 2.15. Histological Analysis

We dissected the skull and removed the brain tissue and soft tissues in the skull base. The samples were then fixed in 10% formalin for 7 days, further decalcified by incubation in ethylenediaminetetraacetic acid (EDTA) solution for 15 days, and finally embedded in paraffin wax. Specimens were sagittally resected into 5 *μ*m-thick slices and stained with hematoxylin and eosin (HE) to distinguish the bone and soft tissues, especially regenerative bone. Digital images of each slide were visualized and captured using a transmission and polarized light Axioskop microscope, Olympus BX51 (Olympus Corp, Tokyo, Japan).

### 2.16. Statistical Analysis

All data in the current study were shown as the mean ± SD of different numbers of independent experiments. Data comparison among different groups was performed using the Student *t*-test or one-way analysis of variance (ANOVA) in SPSS (version 20, Chicago, IL, USA), and *p* < 0.05 was deemed as statistical significance.

## 3. Results

### 3.1. IR Induces Cellular Apoptosis and Intracellular ROS Production

**Figure 1:**
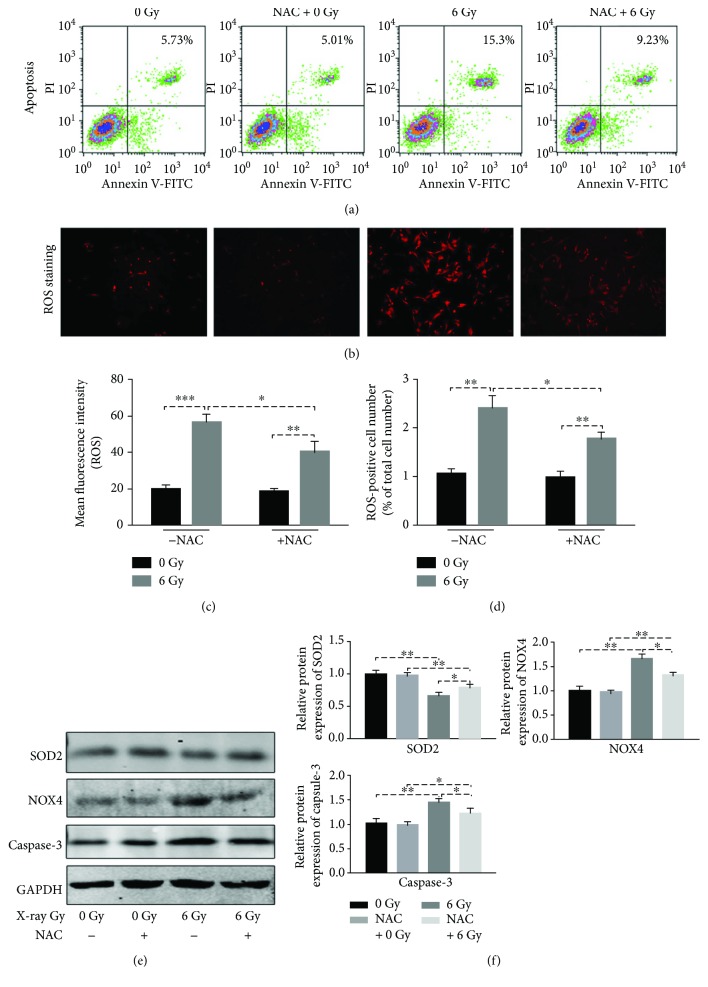
Cellular apoptosis and ROS generation following IR (0 or 6 Gy) in rBMSCs. N-Acetyl-L-cysteine (NAC) is a classical antioxidant, which was used to testify the relationship of ROS generation with apoptosis in our experiment. (a) Cellular apoptosis was measured at 24 h postradiation by using the Annexin V-FITC method. IR-induced apoptosis can be partly rescued by pretreatment with NAC (*n* = 3). (b–d) Detection of ROS level at 24 h postradiation, (b) ROS staining with Fluorometric Intracellular ROS Kit. (c) Mean fluorescent intensity was measured at 640/675 nm. (d) The ROS positive ratio (%) is shown as a percentage of the positive cells to the total cell number (*n* = 3, ^∗^ *p* < 0.05, ^∗∗^ *p* < 0.01, and ^∗∗∗^ *p* < 0.001). (e) Western blot showed the expression level of SOD2, NOX4, and Caspase-3 following IR; GAPDH served as endogenous reference. (f) The densitometric intensities were quantified using Photoshop. All bar graphs were shown as means ± SEM (*n* = 3, ^∗^ *p* < 0.05, ^∗∗^ *p* < 0.01, and ^∗∗∗^ *p* < 0.001).

rBMSCs treated with 6 Gy radiation had a much higher apoptotic ratio than the 0 Gy group (15.3 ± 2.67% versus 5.73 ± 1.19%) (*p* ≤ 0.001) (Figure 1(a)). Subsequently, we investigated the intracellular ROS level of rBMSCs following X-ray exposure. Irradiation increased the ROS production of rBMSCs, which was verified by ROS-positive cell number and mean fluorescence intensity (Figures 1(b)–1(d))). Meanwhile, upregulation of NOX4 and downregulation of SOD2 after radiation also proved this ROS change (Figures 1(e) and 1(f))). Since radiation induced both cellular apoptosis and ROS production, we attempted to reveal the relationship between apoptosis and intracellular ROS.

### 3.2. IR-Induced Cellular Apoptosis Partly Contributed to the ROS Generation

N-Acetyl-L-cysteine (NAC) is a classical antioxidant, which performs its function on the basis of a sulfhydryl group. Pretreatment with NAC significantly reduced the mean fluorescence intensity by 29% and the ROS-positive cell number by 26.7% in irradiated rBMSCs. Similarly, upregulation of SOD2 and downregulation of NOX4 were also observed in the group with NAC pretreatment. However, this ROS inhibition was not found in nonirradiated cells. These data proved the antioxygenation of NAC in the radiation model of rBMSCs. Subsequently, we tried to validate whether this antioxygenation had a role in the inhibition of apoptosis of rBMSCs induced by irradiation. As shown in Figure 1(a), the apoptotic ratio decreased from 15.3 ± 2.67% to 9.23 ± 1.89% (*p* ≤ 0.01) with NAC pretreatment following radiation, and this reduction was also observed in the apoptotic protein expression of Caspase-3, an important marker of apoptosis. However, this cell viability promotion was not seen in nonirradiated cells. These results demonstrated that irradiation-induced cellular apoptosis may be partly due to ROS production stimulated by X-rays.

### 3.3. IR Induces the miR-22 Expression and Efficiency of Lentivirus Transfection in rBMSCs

**Figure 2:**
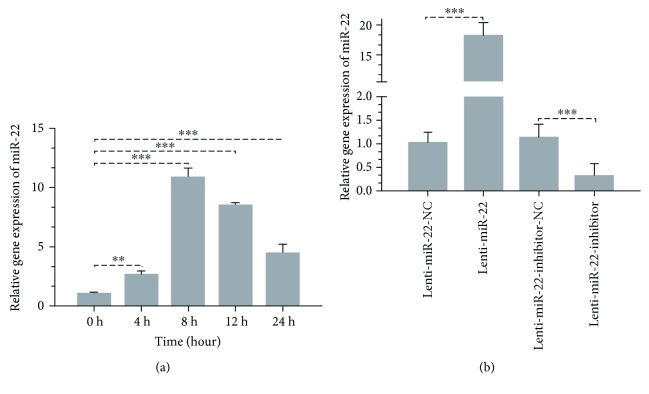
(a) miR-22 expression in rBMSCs following 6 Gy IR and transfection with miR-22 lentivirus. Upregulation of miR-22 showed a time-dependent manner with a peak induction at 8 h (*n* = 3, ^∗∗^ *p* < 0.01 and ^∗∗∗^ *p* < 0.001). (b) RT-PCR verification of miR-22 lentivirus transfection (*n* = 3, ^∗∗∗^ *p* < 0.001).

In an effort to explore the miR-22 expression change upon 6 Gy radiation, rBMSC samples were collected at different time points and detected by means of RT-PCR. A significant increase in the intracellular expression of miR-22 stimulated by radiation was observed in a time-dependent manner. This upregulation reached a peak at 8 h after radiation and lasted 24 h postradiation (Figure 2(a)). In addition, we probed into the expression of miR-22 following different dosages of X-ray radiation at 8 h post-IR (the peak time point), with the result that significant upregulation of miR-22 was detected among different groups (Figure S1). MiR-22 was reported to participate in ROS production through induction by butyrate, myocardial ischemia/reperfusion (I/R), and UV radiation. These investigations indicated that miR-22 may also have a regulatory role on the ROS production and apoptosis via induction by X-ray radiation.

To verify this hypothesis, we constructed a lentivirus of miR-22 (overexpression or downregulation) to perform a gain-and-loss experiment. This efficiency was also proven through RT-PCR analysis (18.235-fold upregulation and 0.311-fold downregulation, *p* ≤ 0.001) (Figure 2(b)). As shown in Figure S2, fluorescence microscopy demonstrated positively stained cells with GFP emission in different groups at 24 h posttransfection. The transfection efficiency of rBMSCs at MOI of 100 was greater than 95.0% in four groups.

### 3.4. miR-22 Overexpression Increased the Intracellular ROS Level

**Figure 3:**
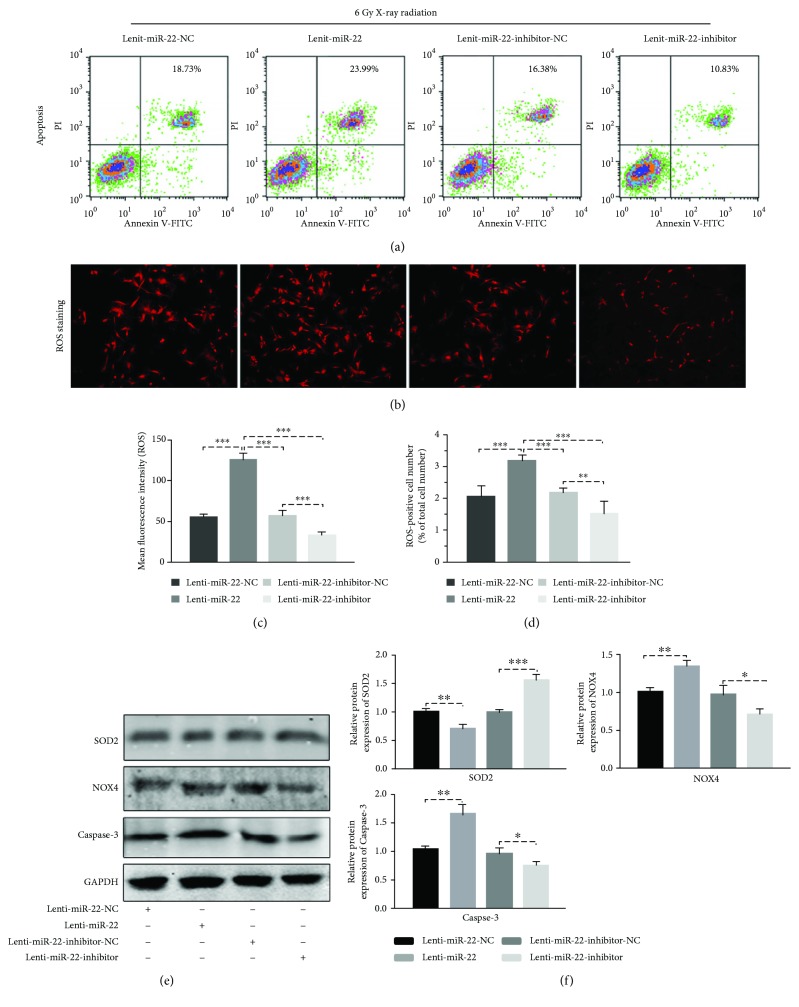
Cellular apoptosis and ROS generation in miR-22-modified rBMSCs following 6 Gy IR. After transfection of miR-22 lentivirus with 48 h, cells were than exposed to IR and samples were collected at 24 h postradiation. (a) Overexpression of miR-22 enhanced the apoptotic ratio of rBMSCs (*n* = 3). (b–d) Inhibition of miR-22 helps cells against the production of ROS. (b) ROS staining. (c, d) Mean fluorescent intensity and the ROS-positive ratio (%) (*n* = 3, ^∗∗^ *p* < 0.01 and ^∗∗∗^ *p* < 0.001). (e) Protein level of SOD2, NOX4, and Caspase-3 in miR-22-modified cells following IR. (f) The densitometric intensity analysis. Both showed that miR-22 played an important role on IR-induced ROS generation and apoptosis, vice versa. All bar graphs were shown as means ± SEM (*n* = 3, ^∗^ *p* < 0.05, ^∗∗^ *p* < 0.01, and ^∗∗∗^ *p* < 0.001).

Twenty-four hours after lentivirus transfection, rBMSCs were exposed to 6 Gy IR. The ROS level was estimated at 24 h postradiation using the methods mentioned above. The results showed that overexpression of miR-22 promoted the ROS release through both ROS-positive cell number (elevated 1.55-fold) and mean fluorescence intensity (elevated 2.3-fold), while cells transfected with the lentivirus miR-22 inhibitor had the capability to reverse the ROS regeneration (Figures 3(b)–3(d)). This regulated role of miR-22 in ROS intervention was further confirmed through detection of ROS-related protein expression (decreased by 25.6% in SOD2 and increased by 31.4% in NOX4) (Figures 3(e) and 3(f)).

### 3.5. Postradiation Survival of rBMSCs Was Rescued by Inhibition of miR-22

We have verified that miR-22 participated in the regulation of IR-induced ROS production. To investigate whether ROS-mediated cellular apoptosis occurs through miR-22, the transfected cells were exposed to IR and further detected using flow cytometry at 24 h postradiation. The results elucidated a positive correlation between the overexpression of miR-22 and cellular apoptosis (Figure 3(a)). Furthermore, the expression of Caspase-3 increased 1.64-fold (*p* < 0.01) in the Lenti-miR-22 group and decreased 0.215-fold (*p* < 0.05) in the Lenti-miR-22-inhibitor group compared to NC-transfected cells (Figures 3(e) and 3(f)). These data confirmed the regulatory role of miR-22 on ROS-mediated cellular apoptosis of rBMSCs induced by IR.

### 3.6. Irradiation Impairs the Osteogenic Differentiation of rBMSCs

**Figure 4:**
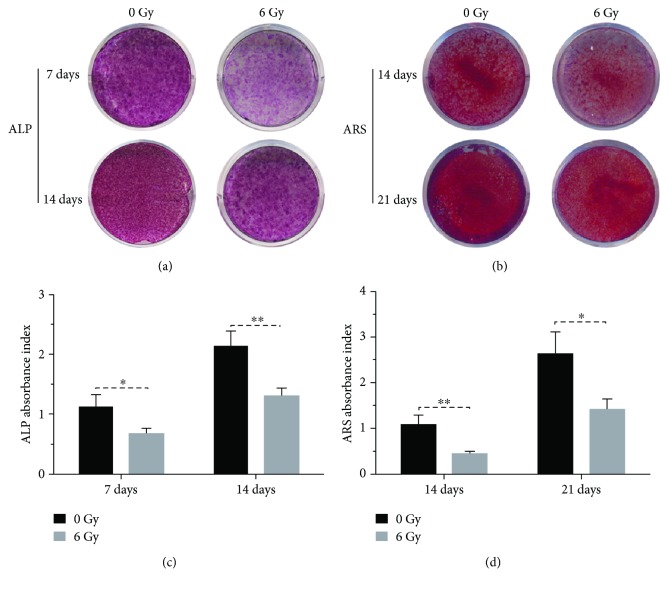
Impaired osteogenic capacity of rBMSCs with IR exposure. (a) ALP staining on days 7 and 14. (b) ARS staining on days 14 and 21. (c, d) The semiquantitative analysis of ALP and ARS staining (*n* = 3,^∗^ *p* < 0.05 and ^∗∗^ *p* < 0.01).

The osteogenic capacity of rBMSCs following X-ray treatment was assessed by ALP/ARS staining and quantification. As shown in Figure 4, ALP staining on day 7 and day 14 indicated a lower density in the irradiated (6 Gy) and subsequent osteogenically differentiated rBMSCs (Figure 4(a)). This qualitative evaluation was further confirmed by semiquantitative analysis using absorbance detection, which revealed that ALP activity in the control group was 1.94-fold (*p* < 0.05) and 1.65-fold (*p* < 0.01) higher than that in the irradiated group on days 7 and 14, respectively (Figure 4(c)). A similar phenomenon was seen in ARS assessment; a decrease in the density of mineralized deposits and nodules was seen in the irradiated group at both day 14 and day 21 (Figure 4(b)). Absorbance from calcium deposit staining was significantly lower in the 6 Gy sample than that in the 0 Gy group at different time points (Figure 4(d)). These data demonstrated a more pronounced osteogenic property in nonirradiated rBMSCs compared to that with 6 Gy exposure. This in vitro model revealed that osteogenic differentiation of rBMSCs was significantly inhibited by X-ray exposure with a dosage of 6 Gy.

### 3.7. Reverse of IR-Induced Apoptosis by miR-22 Inhibitor Promoted the Osteogenic Capability of rBMSCs In Vitro

**Figure 5:**
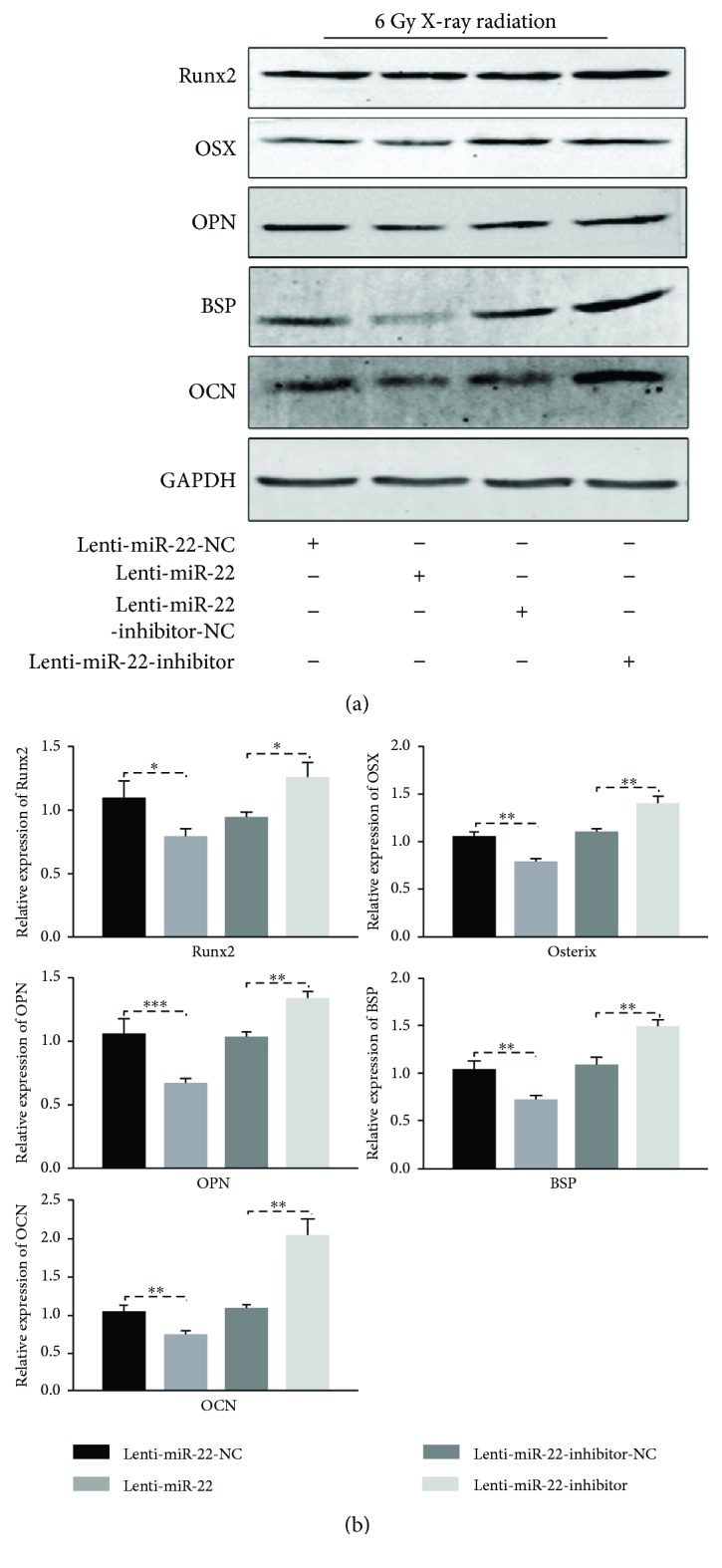
The protein level of osteogenic related markers (Runx2, Osx, OPN, BSP, and OCN) in miR-22-modified rBMSCs with 6 Gy IR exposure. (a) Protein bands. (b) The densitometric intensity analysis (normalized to GAPDH) (*n* = 3, ^∗^ *p* < 0.05, ^∗∗^ *p* < 0.01, and ^∗∗∗^ *p* < 0.001).

**Figure 6:**
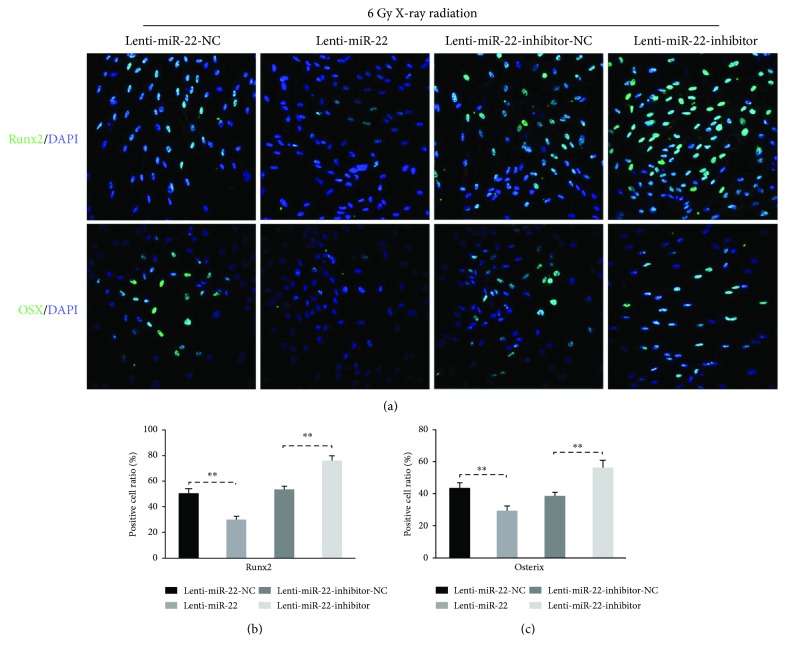
Immunofluorescence analysis of bone-specific markers (Runx2 and Osterix) in miR-22-modified rBMSCs with IR treatment. (a) Overexpression of miR-22 aggravated the osteogenic impairment induced by IR. (b, c) The ratio of immunoreactive cells was calculated by dividing the number of positive cells by the number of total cells stained with DAPI. We counted 800–1000 cells in random fields under microscopy for each group (*n* = 3, ^∗∗^ *p* < 0.01).

To elucidate whether miR-22-mediated ROS-dependent apoptosis has a regulatory role on the osteogenesis of rBMSCs following IR exposure, transfected cells following IR were cultured in osteoinductive medium for 14 and 21 days. The protein expression level of osteogenic markers (Runx2, OSX, OPN, BSP, and OCN) were measured using Western blot. As shown in Figures 5(a) and 5(b), these markers were repressed in the Lenti-miR-22 group but significantly promoted in the Lenti-miR-22-inhibitor group in comparison with the Lenti-miR-22-NC or Lenti-miR-22-inhibitor-NC group, respectively. Furthermore, cellular immunofluorescence was performed in 4 groups with osteogenic induction for 14 days to test the expression of Runx2 and Osterix (Figure 6). The results showed that 50.06 ± 3.97% of Lenti-miR-22-NC-tansduced cells and 53.17 ± 2.86% of Lenti-miR-22-inhibitor-NC-tansduced cells expressed Runx2, respectively. This expression percentage was much lower (29.46 ± 3.26%, *p* < 0.01) in the Lenti-miR-22 group and dramatically higher (75.43 ± 4.45%, *p* < 0.01) in the Lenti-miR-22 inhibitor group. The positive expression ratio of Osterix in four groups (42.87 ± 3.84% in the Lenti-miR-22-NC group, 29.0 ± 3.38% in the Lenti-miR-22 group, 40.9 ± 2.2% in the Lenti-miR-22-inhibitor-NC group, and 57.0 ± 3.2% in the Lenti-miR-22-inhibitor group) had a similar pattern as the expression of Runx2.

**Figure 7:**
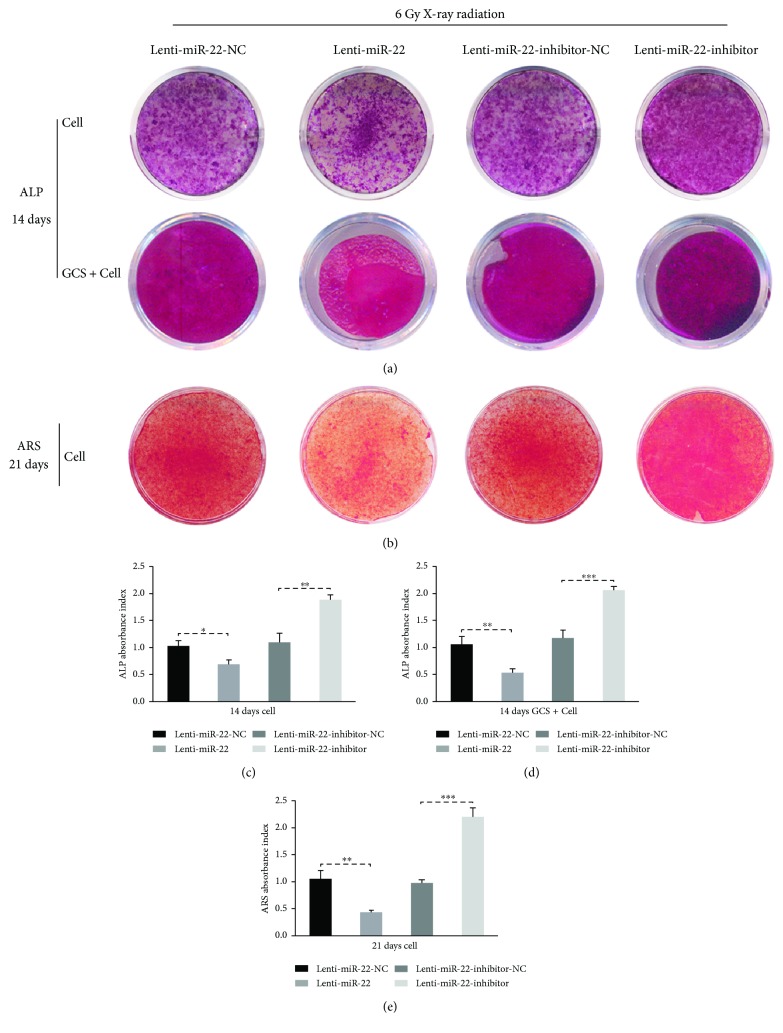
miR-22 negatively regulated the osteogenic capability of irradiated rBMSCs. (a) ALP staining of miR-22-modified cells and GCS scaffold combined with cells on day 14. (b) ARS staining of miR-22-modified cells on days 21. (c, d) The semiquantitative analysis of ALP in cell samples and GCS scaffold combined with cells samples. (e) The semiquantitative analysis of ARS in cell samples (*n* = 3, ^∗^ *p* < 0.05, ^∗∗^ *p* < 0.01, and ^∗∗∗^ *p* < 0.001).

ALP staining of 14-day samples was significantly attenuated in the Lenti-miR-22-transfected and irradiated groups but dramatically promoted in the Lenti-miR-22-inhibitor group (Figure 7(a)). This trend was also observed in ARS staining of 21-day-old samples (Figure 7(b)). The semiquantitative analysis of ALP showed a 33.3% lower expression level in the Lenti-miR-22-transfected and irradiated groups and a 73.3% higher expression level in the Lenti-miR-22-inhibitor group than in the Lenti-miR-22-NC and Lenti-miR-22-inhibitor-NC groups, respectively (Figures 7(c) and 7(d)). Similarly, the semiquantitative analysis of ARS staining was consistent with the trend of ALP staining (Figure 7(e)). To summarize, these findings, including protein expression, immunofluorescence, and ALP/ARS staining, demonstrated that miR-22-mediated ROS-dependent apoptosis has a regulatory role on the osteogenesis of rBMSCs following IR exposure.

### 3.8. In Vivo Estimation of Bone Regeneration by miR-22 Transfection and following Radiation

**Figure 8:**
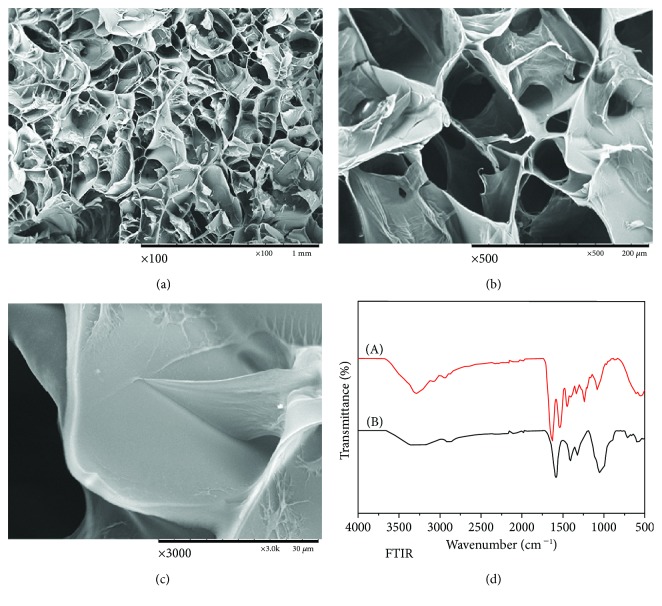
Evaluation of the GCS scaffold. (a–c) Surface structures of GCS were captured by SEM with different magnifications (×100, ×500, ×3000). (d) FTIR analysis. (A) Gelatin and (B) CMC (*n* = 3).

**Figure 9:**
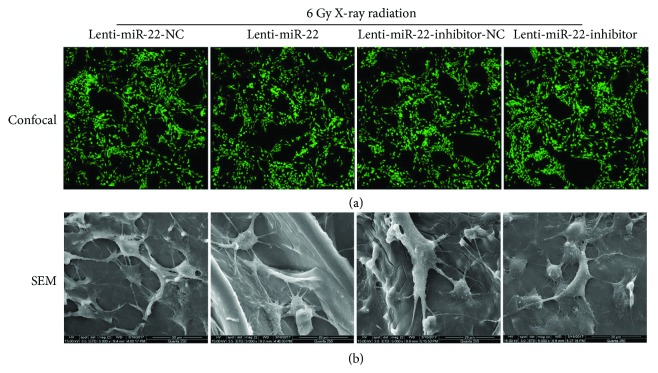
Biocompatibility and adhesion property of scaffold. (a) CLSM images showed a favorable cell morphology and proliferation of miR-22-modified rBMSCs (*n* = 3). (b) Cell adhesion was captured by using SEM scanning (*n* = 3).

To evaluate the osteogenic capacity of rBMSCs with miR-22 transfection and following radiation, we fabricated the scaffold material composed of gelatin and carboxymethyl chitosan (CMC) by using the method of freeze-drying. SEM showed the microstructure of the G-CMC scaffold with different magnifications (Figures 8(a)–8(c)). FTIR analysis verified the existence of two different components (Figure 8(d)). After transfection with miR-22 lentivirus and following IR, rBMSCs were seeded onto the G-CMC scaffold that was presterilized for 7 days and then stained with calcein AM for 15 min. Confocal microscopy showed a sufficient surface of the G-CMC scaffold for cell attachment and proliferation. There was no significant difference in cell number and morphology among the 4 groups with genetic modification (Figure 9(a)). A similar phenomenon was observed in SEM detection, which showed cell spreading (Figure 9(b)).

To validate whether miR-22 modified rBMSCs with subsequent IR exposure showed a distinct capacity of bone regeneration in vivo, we established a bilateral calvarial defect (5 mm) model in female rats and implanted the compounds, including rBMSCs/Lenti-miR-22-NC/IR/G-CMC (group 1), rBMSCs/Lenti-miR-22/IR/G-CMC (group 2), rBMSCs/Lenti-miR-22-inhibitor-NC/IR/G-CMC (group 3), rBMSCs/Lenti-miR-22-inhibitor/IR/G-CMC (group 4), into the defect areas. Experimental compounds were placed into the right defect and compared with G-CMC alone, which was placed on the left site.

**Figure 10:**
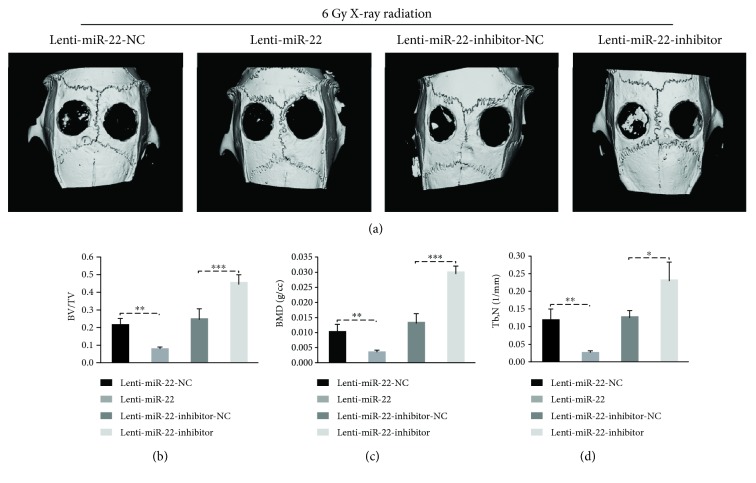
Micro-CT evaluation of the osteogenic capacity of miR-22-modified and irradiated rBMSCs in vivo. (a) Images with three-dimensional reconstruction revealed different restorative effects among four groups (*n* = 6). (b, c, d) Quantitative analysis, new bone volume relative to tissue volume (BV/TV), the bone mineral density (BMD) and trabecular number (Tb.N) (*n* = 6, ^∗^ *p* < 0.05, ^∗∗^ *p* < 0.01, and ^∗∗∗^ *p* < 0.001).

**Figure 11:**
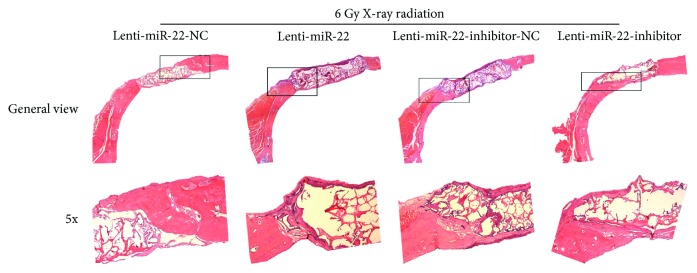
Histological analysis of the reparative effect among different groups. Samples were cut in coronal section to make HE slides (*n* = 3).

The new bone formation in the defect area was measured using micro-CT at 8 weeks after the surgical procedure, which showed newly formed bone within the G-CMC area. As shown in Figure 10(a), group 2 produced a lower bone formation, and group 4 had significantly higher bone formation than group 1 and group 3, respectively. There was no obvious newly bone formation in G-CMC only group (right site in the figure). The quantitative analysis of newly formed bone tissue was performed using micro-CT assessment. The BV/TV in group 2 (6.0 ± 1.0%) was much lower than that in group 1 (13.3 ± 3.05%), while this ratio was dramatically higher in group 4 (40.0 ± 3.6%) than that in group 3 (18.0 ± 4.0%) (Figure 10(b)). Moreover, the BMD in group 2 (0.076 ± 0.016 g/cm^3^) and group 4 (0.33 ± 0.026 g/cm^3^) also had the same trend with the BV/TV compared to that in group 1 (0.133 ± 0.015 g/cm^3^) and group 3 (0.156 ± 0.15 g/cm^3^) (Figure 10(c)). The similar trend was also seen in Tb.N (Figure 10(d)). Thus, miR-22 overexpression may facilitate the osteogenic damage caused by IR exposure, while miR-22 inhibition seemed to have a role on the reverse of this biological damage. In addition, skull samples were fixed and cut in coronal direction to make HE slides, and this observation also supported the findings from micro-CT or in vitro study (Figure 11).

## 4. Discussion

Ionizing radiation (IR) characterized by X-ray exposure is frequently performed for medical applications. The typical influence of IR exposure on cells is composed of single- or double-strand DNA breaks, chromosomal destruction, cell cycle arrest, impairment of stemness (differentiation capacity), production of ROS, and even cell death [14]. In the current study, we demonstrated that miR-22-mediated ROS regeneration had an important regulatory effect on cellular apoptosis and further osteogenic differentiation following IR exposure. We designated 6 Gy IR as the experimental treatment group and 0 Gy as the control group. The utilization of 6 Gy had an influence on DNA damage, cell cycle, and stemness, but no significant inhibition of the proliferation rate was proven through the colony formation assay [13].

Radiation, as well as other exogenous physical or chemical agents, can induce the production of ROS. This accumulation may lead to excessive oxidative stress and DNA damage, which are deemed as the main underlying mechanisms inducing cell injury. At 24 h after 6 Gy IR, increased intracellular ROS level was verified by ROS staining and mean fluorescence intensity measurements. The findings were consistent with the elevation of ROS in human keratinocyte HaCat cells at 24 h after X-ray radiation [31]. NOX4 belongs to the NOX/DUOX family and participates in the production of ROS in a wide range of cell types [32]. Superoxide dismutase 2 (SOD2) belongs to the iron/manganese superoxide dismutase family and allows cells to scavenge intracellular ROS. Furthermore, SOD2 also has an important role against cellular apoptosis induced by ROS, IR, and inflammatory cytokines [33]. Through establishment of a total body irradiation mouse model, scholars found that ROS regeneration was intimately correlated with the upregulation of NOX4 and downregulation of SOD2 in bone marrow injury [34]. This differential expression of NOX4 and SOD2, along with the elevation of ROS, was also proven in our in vitro model of rBMSCs. To summarize, 6 Gy IR can induce the generation of ROS in rBMSCs.

IR-induced BMSC apoptosis has been extensively studied, but without coherent conclusion [4, 7–10, 13, 34–37]. We hold the view that the radiosensitivity of MSCs may depend to a large extent on several factors, such as the derivation of MSCs (bone or mesenchymal tissue), radiation resource (X-ray, *γ-*ray, or UV), dosage and dose rate, biological status of the cell at the time of IR, and the detection time (early or late). Rat BMSCs in our IR model showed a moderate radiosensitivity proven by the increase in the apoptotic ratio at 24 h postradiation both in Annexin V-FITC and Caspase-3 protein detection. To verify whether IR-induced ROS production participated in the regulation of IR-mediated apoptosis, we performed the intervention trial with N-acetyl-L-cysteine (NAC), a type of membrane-penetrating antioxidant that is widely used in the elimination or abatement of intracellular ROS. Through pretreatment with NAC for 2 h, a significant reduction in ROS level was detected following IR compared with the control group. Moreover, the inhibition of ROS had a protective effect on IR-induced apoptosis. This negative correlation of ROS and apoptosis was consistent with the findings of Banerjee et al., who investigated the IR-induced cell death and oxidative stress in mouse embryonic fibroblasts (MEFs) [38]. The initiation of apoptosis is mediated by the caspase family, and ROS can activate these enzymes through mitochondria injury, cytochrome c release, and other signaling pathway activation.

MicroRNAs (miRNAs) have a negative regulatory role on mRNA through degradation or inhibition of posttranslation via binding to the 3′-untranslated region (3′-UTR) of target mRNAs and have been verified as multifunctional genes involved in cell cycle, survival and death, proliferation, differentiation, and so on. Researchers have found that miRNA response following radiation may participate in the biological process of DNA damage, cell cycle, or proliferation. For this reason, more studies have focused on miRNA expression analysis through miRNA array profiles upon UV and IR exposure in different cell types, and they found that miR-22 was upregulated with statistically significant differences [20–24]. In our study, we also showed the upregulation of miR-22 following IR at different time points within 24 hours. miR-22 has been reported to participate in ROS-mediated apoptosis induced by butyrate, contributes to myocardial ischemia-reperfusion injury by affecting the mitochondrial function (ROS, ATP, and membrane potential), and has a neuroprotective role in a 6-hydroxydopamine-induced model of Parkinson's disease via regulation of ROS [28–30]. The role of miR-22 in ROS regeneration was not constant, and it may be involved in activation or inhibition according to the cell type and different stimuli. The issue of whether miR-22 is an indispensable regulator of ROS and apoptosis in rBMSCs following IR has not been elucidated. In the current research, we verified that the overexpression of miR-22 can enhance the ROS level and apoptotic ratio induced by IR and vice versa. Through this gain and loss analysis, we confirmed that IR-induced miR-22 expression participated in the process of ROS-mediated cellular apoptosis.

rBMSCs belong to the multipotential stem cell family and hold the capability to differentiate into multilineage cells, including osteoblasts, adipocytes, chondrocytes, and fibroblasts. Although IR, especially for X-rays, is deemed as a mainstream therapeutic method for malignancies, its side effects, such as decreased viability of bone tissues, osteomyelitis, osteoradionecrosis, and even IR induced osteosarcoma, cannot be ignored. In the current investigation, we focused on the osteogenic influence of rBMSCs following IR and its relationship with miR-22-mediated ROS production and subsequent apoptosis. Previous publications have proven the impairment of osteogenesis of MSCs following IR in a dose-dependent manner [39–41]. In our findings, 6 Gy IR was sufficient to impair the osteogenic capacity of rBMSCs, which was proven by ALP/ARS analysis in vitro. BMSCs with higher resistance to apoptosis hold higher osteogenic potential. Wang et al. showed that the inhibition of dexamethasone-induced cytotoxicity and subsequent cellular apoptosis were dependent on the autophagy targeting the mTOR pathway, and this protection of cell viability correlated with new bone formation. In addition, BMSCs from the novel bone group showed a lower ratio of cell apoptosis and higher proportion of autophagy [42]. This relationship between apoptosis and osteogenic capacity was also verified in our findings. Oxidative stress induced by H_2_O_2_ has been linked to the decreased potential of osteogenic differentiation in MSCs by affecting the transcriptional programs and promoting the adipogenic differentiation [43]. However, the mechanism of ROS-mediated osteogenic impairment was undefined. With our verification, we suggest that IR-induced damage of osteogenesis may be partly due to ROS-mediated cellular apoptosis, which was regulated by the overexpression of miR-22.

As for the in vitro analysis of the impact of miR-22-mediated ROS and apoptosis on osteogenesis, we measured the ALP/ARS staining, protein expression, and immunofluorescence. To validate this regulatory function, we fabricated a gelatin/carboxymethyl chitosan scaffold for cell seeding and subsequent in vivo evaluation. Gelatin was chosen for its biocompatibility and fabrication capabilities, as it has been widely used to construct a 3D scaffold for bone regeneration. Carboxymethyl chitosan is a derivation of chitosan and demonstrates better water solubility and adhesive capacity than normal chitosan, and thus, it could be used for controlled release experiment and further tissue engineering [44]. Because we focused on the osteogenic capability of miR-22-modified rBMSCs following IR, this scaffold was suitable for the current study design in terms of its good biocompatibility, cell adhesive capacity (proved by SEM and confocal microscopy), and most importantly, low promotion of osteogenesis for the scaffold alone. In vivo analysis, including the micro-CT and HE slide observation, also demonstrated that miR-22-modified rBMSCs following IR hold the same trend as that detected in vitro.

In conclusion, IR was an important exogenous stimulus to induce the production of ROS and further apoptosis in rBMSCs, and these biological processes may be conducted by the overexpression of miR-22. Furthermore, miR-22-mediated ROS regeneration and apoptosis were intimately correlated with the impairment of osteogenic capacity both in vitro and in vivo. However, the mechanism of miR-22 in the regulation of ROS and apoptosis was still unclear. Further study should be performed to determine which mechanism dominates the ROS production, such as mitochondrial pathway, death receptor pathway, or ER pathway, as well as to elucidate the role of autophagy on this biological process.

## Supplementary Materials

Supplementary Materials Figure S1: miR-22 expression in rBMSCs following different dosages of X-ray radiation at 8 h post-IR (*n* = 3, ^∗∗∗^ *p* < 0.001). Figure S2: rBMSCs were transfected with miR-22 lentivirus. Positive cells with GFP label were captured under fluorescence microscopy, and transfect efficiency was greater than 95% in each group (*n* = 3).
